# A new species of *Coccus* (Hemiptera, Coccoidea, Coccidae) from China

**DOI:** 10.3897/zookeys.244.4045

**Published:** 2012-11-22

**Authors:** Fang Wang, Ji-Nian Feng

**Affiliations:** 1 Key Laboratory of Plant Protection Resources and Pest Management, Ministry of Education, Entomological Museum, College of Plant Protection, Northwest A&F University, Yangling, Shaanxi Province, 712100, China.

**Keywords:** Hemiptera, Coccoidea, soft scale, new species, China

## Abstract

A new species of soft scale, *Coccus multisetus* Wang & Feng, **sp. n.** is described and illustrated from Yunnan, China. A key to adult females of all *Coccus*known from China is provided.

## Introduction

The Coccidae or soft scales (Hemiptera: Sternorrhyncha: Coccoidea) is the third largest family of the Coccoidea after the Diaspididae (armored scales) and the Pseudococcidae (mealybugs) (Ben-Dov et al. 2012). Soft scales are widespread throughout the world and many of them are important pests on agricultural and horticultural crops and ornamental plantings (Henderson and Hodgson 2005), such as *Ceroplastes rubens* Maskell, *Ceroplastes japonicus* Green and *Didesmococcus koreanus* Borchsenius. Nonetheless, some species are very beneficial to man, such as *Ericerus pela* (Chavannes), whose wax provides an important raw material for many industries (Tang 1991).

*Coccus* is the oldest genus within the Coccidae (Avasthi and Shafee 1991). The genus was proposed by Linnaeus in 1758, with *Coccus hesperidum* Linnaeus as its type species, and belongs to the tribe Coccini, subfamily Coccinae (Hodgson 1994). *Coccus* is a very large genus present in all zoogeographical regions (Hodgson 1994) but is in urgent need of revision. Hitherto, coccidologists have listed about 94 species in this genus (Williams and Ben-Dov 2009, Martin and Lau 2011, Ben-Dov et al. 2012), of which 10 have been recorded from China (Takahashi 1932, Tao et al. 1983, Tang 1991, Martin and Lau 2011). Moreover, some *Coccus* species are pests of horticultural and ornamental plants in China (Yang 1982).

In this paper, we describe and illustrate the adult female of a new species, *Coccus multisetus* Wang & Feng, sp. n. This new species shares certain characteristics with *Ceroplastes formicarii* (Green), which has previously been placed in *Taiwansaissetia* Tao et al., 1983, but that genus has now been synonymised with *Coccus* (Lin et al. in press). A key is provided for separation of the 11 species of *Coccus* currently known from China.

## Materials and methods

The specimens were all immersed in 75% ethanol, and then prepared and mounted mainly according to the method of Hodgson and Henderson (2000). The terminology used in the description is mainly that of Hodgson (1994). Characters were examined under a Nikon compound microscope. An Olympus BH-2 stereoscopic microscope was used for drawing. The illustrations show adult female specimens, with the dorsum depicted on the left side and the venter on the right side, with enlargements of important characters shown around the main illustration. All measurements were made using the software NIT-Elements D and were given in micrometers (µm) or millimeters (mm).

All specimens are deposited in the Entomological Museum of Northwest A&F University, Yangling, Shaanxi, China (NWAFU).

## Taxonomy

### Genus Coccus

Linnaeus, 1758

http://species-id.net/wiki/Coccus

Coccus Linnaeus, 1758: 455–457. Type species: *Coccus hesperidum* Linnaeus, 1758.

#### Generic diagnosis.

**Adult female.** Body oval, elongate or pyriform; usually flat, moderately convex, or nearly hemispherical. **Dorsum.** Derm membranous; gradually increasing in thickness during ageing. Dorsal setae setose, spinose, clavate or cylindrical. Dorsal tubular ducts and dorsal submarginal tubercles present or absent. Preopercular pores present or absent, varying in number and shape. Anal plates together quadrate. Anal ring with 6 or 8 setae. **Margin.** Marginal setae simple to branched; stigmatic clefts deep or shallow, each with 2–8 stigmatic spines. **Venter.** Antennae 2–8 segmented. Legs well developed or reduced; with or without a tibio-tarsal articulatory sclerosis. Spiracular disc-pores with 5 loculi. Pregenital disc-pores mainly with 10 loculi, present around anal opening, or extending anteriorly, even as far as head. Ventral tubular ducts present or absent; when present located medially, or forming a sparse or dense submarginal band.

#### Key to adult female *Coccus* known from China

**Table:** 

1	Body elongate, tapering at both anterior and posterior ends; antennae 6 segmented; with a dense submarginal band of ventral tubular ducts	*Ceroplastes takanoi* Takahashi
–	Characters not in the above combination	2
2	Dorsal submarginal tubercles present	3
–	Dorsal submarginal tubercles absent	9
3	Antennae 2 segmented; interantennal setae absent	*Ceroplastes cambodiensis* Takahashi
–	Antennae 6–8 segmented; interantennal setae present	4
4	With only 1 pair of pregenital setae	*Ceroplastes capparidis* (Green)
–	With 3 pairs of pregenital setae	5
5	Ano-genital fold with 4 pairs of setae along anterior margin	*Ceroplastes longulus* (Douglas)
–	Ano-genital fold with 2 pairs of setae along anterior margin	6
6	Dorsal tubular ducts present; the outer ductules of dorsal tubular ducts very wider than those on venter	*Ceroplastes moestus* De Lotto
–	Dorsal tubular ducts present or absent; if present, the outer ductules of dorsal tubular ducts narrower than those on venter	7
7	Marginal setae mostly fine and not branched	*Ceroplastes hesperidum* L.
–	Marginal setae mostly branched	8
8	Body elongate oval; ventral tubular ducts present medially between the middle and hind legs	*Ceroplastes viridis* (Green)
–	Body broadly oval; ventral tubular ducts sparsely scattered over venter, and not restricted to the median area	*Ceroplastes discrepans* (Green)
9	Dorsal setae spinose	*Ceroplastes pseudomagnoliarum* (Kuwana)
–	Dorsal setae setose	10
10	With 3 pairs of pregenital setae; without a submarginal band of ventral tubular ducts	*Ceroplastes formicarii* (Green)
–	With 2 pairs of pregenital setae; with a sparse submarginal band of ventral tubular ducts	*Ceroplastes multisetus* sp. n.

### Coccus multisetus

Wang & Feng sp. n.

urn:lsid:zoobank.org:act:F00857A0-3484-4537-96EC-AD2C10B67F15

http://species-id.net/wiki/Coccus_multisetus

Figure 1

#### Material examined.

**Holotype:** adult female. CHINA, Yunnan Prov., Natural reserve of Mengyang. 17.v.2012, on *Mangifera indica* (Anacardiaceae), Fang Wang (NWAFU).

**Paratypes:** 3 adult females, the data same as holotype.

#### Adult female.

**Unmounted material.** Convex, sometimes nearly hemispherical; dark brown with a pale brown marginal band. Dried materials hard, bearing many ridges from median longitudinal ridge.

#### Mounted material.

Body broadly oval, 2.6–3.6 mm long, 2.0–2.8 mm wide. Anal cleft about 1/7th of body length. Stigmatic clefts distinct.

#### Dorsum.

Derm membranous, with cell-like clear areas. Dorsal setae setose, slender, each 16–30 µm long, with well-developed basal sockets, sparsely distributed over dorsum but absent from median area. Dorsal pores circular, each with a dark rim and about 1–2 µm in diameter, sparsely distributed on dorsum. Dorsal microducts, each with a very short outer ductule and a normal inner filamentous ductule, present in each cell-like area. Dorsal tubular ducts and dorsal submarginal tubercles absent. Preopercular pores, each 3–4 µm in diameter, present in an elongate group anterior to anal plates. Anal plates each broadly triangular, 152–169 µm long, 90–98 µm wide; anterior and posterior margins subequal in length, outer angle nearly a right-angle; each plate with 6 or 7 apical or subapical setae, each 9–16 µm long. Ano-genital fold with 2 pairs of long setae, each 45–60 µm long, present along anterior margin plus 3 pairs of setae, each 28–40 µm long, along each lateral margin. Anal ring subcircular, with 2 or 3 rows of translucent pores and 6 anal ring setae.

#### Margin.

Marginal setae, each 18–29 µm long, setose, fine, straight or curved, all with well-developed basal sockets; with 40–57 setae between anterior clefts, 14–18 setae between each anterior cleft and posterior cleft, and 32–43 setae between each posterior cleft and anal cleft. Stigmatic clefts shallow, each with 3–8 stigmatic spines: with 3 median spines, each 22–38 µm long, blunt, stout, larger than more lateral spines and broadly based; lateral spines, each 8–19 µm long, blunt, smaller and with pointed apices. Eyespots not found.

#### Venter

**.** Derm entirely membranous. Antennae 8 segmented, each 260–284 µm long; third segment longest; with 2 pairs of interantennal setae, each 20–38 µm long. Clypeolabral shield 206–217 µm long, 166–190 µm wide; labium 102–115 µm long, 96–109 µm wide, with 4 setae (24–28 µm) on each side. Legs rather slender; each trochanter with a pair of sensory pores on each side and a single long seta on its ventral surface; each leg with a weak articulation between tibia and tarsus, but with no articulatory sclerosis; tarsal digitules both slender, knobbed and longer than claw digitules, claw digitules both with knobbed apices, but one smaller than the other; claws without a denticle; dimensions of metathoracic leg: coxa 60–75 µm, trochanter+femur 132–160 µm, tibia 80–98 µm and tarsus 68–87 µm. With 2 pairs of long pregenital setae in both segments VI & VII, each 48–66 µm long; submarginal setae present in a single row, each 5–12 µm long; other ventral setae slender, each 6–12 µm long, quite sparsely distributed. Spiracles normal. Spiracular disc-pores each about 4–6 µm in diameter and mainly with 5 loculi; present in a band 2–4 pores wide between stigmatic cleft and each spiracle; with 26–38 pores in each anterior spiracle band and 34–44 pores in each posterior band. Pregenital disc-pores each about 5–7 µm in diameter and mainly with 10 loculi; present around anal opening. Ventral tubular ducts of 2 types: 1) a duct with an elongate outer ductule, each about 15–24 µm long and 2–4 µm wide; an inner ductule about half width of outer ductule, each about 16–24 µm long and 2–3 µm wide; and with a well-developed terminal gland, each about 3–5 µm wide; present medially on head posterior to mouthpart and on prothorax; and 2) a duct with an elongate outer ductule, each about 18–24 µm long and 2–4 µm wide; an inner ductule slightly longer than outer ductule and narrower than that of type 1), each about 20–26 µm long and 1–2 µm wide; and with a well-developed terminal gland, each about 2–3 µm wide; forming a sparse submarginal band and also present medially on meso- and metathorax, extending laterally and mingling with type 1) ducts.

**Figure 1.**
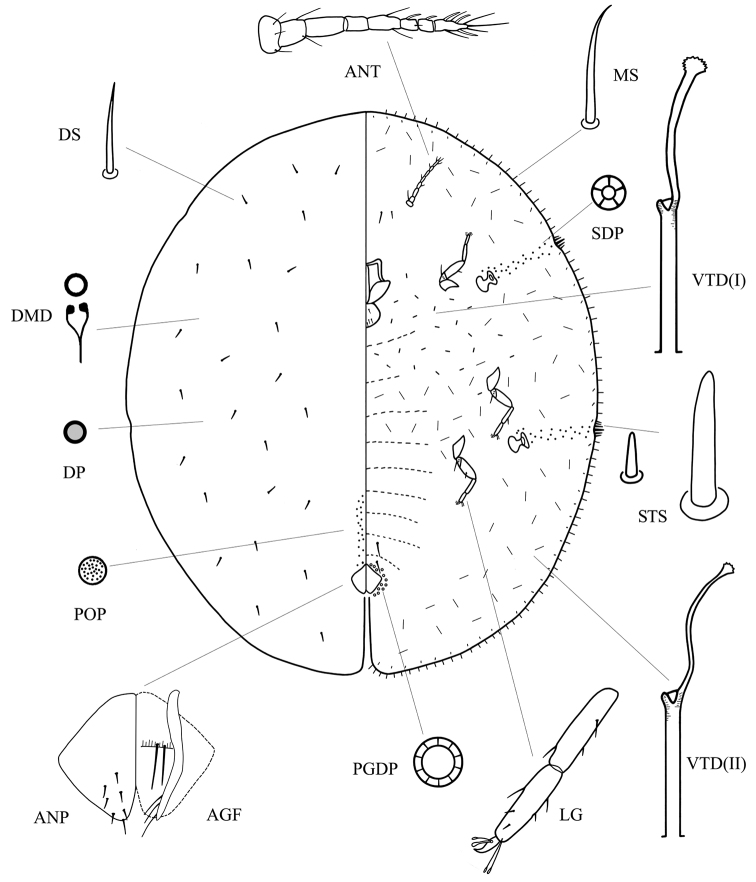
*Coccus multisetus* sp. n. **ANT** antenna; **ANP** anal plate; **AGF** ano-genital fold; **DMD** dorsal microduct; **DP** dorsal pore; **DS** dorsal seta; **LG** tibio-tarsus of hind leg; **MS** marginal seta; **PGDP** pregenital disc-pore; **SDP** spiracular disc-pore; **STS** stigmatic spines; **VTD(I)** ventral tubular duct of type 1); **VTD(II)** ventral tubular duct of type 2).

#### Distribution.

China (Yunnan).

#### Etymology.

The species epithet *multisetus* refers to the many setae on the dorsal surface of the anal plates.

#### Comments.

Adult females of *Ceroplastes multisetus* are superficially similar to those of *Ceroplastes formicarii* (Green), which also had been collected in the nests of ants on *Mangifera indica*. The new species and *Ceroplastes formicarii* (data from Hodgson, 1994, as *Taiwansaissetia formicarii*) share some distinct characteristics: 1) presence of setose dorsal setae; 2) lack of a tibio-tarsal articulatory sclerosis; 3) pregenital disc-pores restricted to around anal opening, and 4) lack of dorsal tubular ducts and submarginal tubercles. These distinct characteristics of the two species differ from those of typical *Coccus*, and might be due to their myrmecophiloushabit and adaptation to living inside ant nests (Lin et al. in press).

However, *Ceroplastes multisetus* can be distinguished by the possession of the following features (character states of *Ceroplastes formicarii* in brackets): 1) 2 pairs of pregenital setae present (3 pairs); 2) 6 or 7 apical or subapical setae on each plate (3 or 4); 3) a submarginal band of ventral tubular ducts (absent); 4) dorsal setae nearly absent on median area (present), and 5) absence of a denticle on the claw (present). Although Hodgson (1994), when studying slide-mounted specimens considered to be *Ceroplastes formicarii*, found their morphology to be rather variable, he noted none of the differences mentioned here, other than the presence of the denticle on the claw. It is thus considered that *Ceroplastes multisetus* is an undescribed species which may be close to *Ceroplastes formicarii*.

## Supplementary Material

XML Treatment for Coccus

XML Treatment for Coccus multisetus
